# Primary care experiences of adults reporting learning disability: a probability sample survey

**DOI:** 10.3399/BJGP.2024.0056

**Published:** 2024-11-12

**Authors:** Samuel J Tromans, Lucy Teece, Rohit Shankar, Angela Hassiotis, Traolach Brugha, Sally McManus

**Affiliations:** Department of Population Health Sciences, University of Leicester, Leicester, and honorary consultant in psychiatry of intellectual disability, Adult Learning Disability Service, Leicestershire Partnership NHS Trust, Leicester.; Department of Population Health Sciences, University of Leicester, Leicester.; University of Plymouth Peninsula School of Medicine, Plymouth, and consultant neuropsychiatrist, Cornwall Partnership NHS Foundation Trust, Truro.; Division of Psychiatry, University College London, London.; Department of Population Health Sciences, University of Leicester, Leicester.; School of Health and Medicine, City St George’s, University of London, London, and affiliated researcher, National Centre for Social Research, London.

**Keywords:** adult, epidemiology, learning disabilities, primary health care, surveys and questionnaire

## Abstract

**Background:**

Adults with learning disability face multiple adversities, but evidence on their needs and primary care experiences is limited.

**Aim:**

To compare the characteristics and primary care experiences of adults reporting learning disability with those who did not.

**Design and setting:**

This was an analysis of the 2022 General Practice Patient Survey, a national probability sample survey conducted in 2022 with people registered with NHS primary care in England.

**Method:**

This analysis reports descriptive profiles, weighted and with 95% confidence intervals. Logistic regression models adjusting for gender, age, ethnicity, and area-level deprivation compared experiences of adults reporting learning disability with those who did not.

**Results:**

Survey participants comprised 623 157 people aged ≥16 years, including 6711 reporting learning disability. Adults reporting learning disability were more likely to be male, younger, of mixed or multiple ethnicities, and live in more deprived areas. All chronic conditions included in the survey were more common in adults reporting learning disability, especially reported sensory, neurodevelopmental, neurological, and mental health conditions. Adults reporting learning disability were twice as likely to have a preferred GP, and less likely to find their practice’s website easy to navigate. They were also less likely to have confidence and trust in their healthcare professional, or feel their needs were met.

**Conclusion:**

Adults reporting a learning disability had a higher likelihood of chronic health conditions. Their reported experiences of primary care indicate that, despite recent initiatives to improve services offered, further adaptations to the consistency and ease of access to primary care is needed.

## Introduction

Learning disability (LD) is defined by the World Health Organization as a neurodevelopmental condition, characterised by significant limitations in intellectual functioning (approximately ≥2 standard deviations below the mean on standardised testing) and adaptive behaviour (conceptual, social, and practical skills), with onset during the developmental period.^1^ There are approximately 1.5 million people with LD in England, representing around 2.5% of the population,^2^ although only a fraction of these people are listed on their general practice’s learning disability register.^3^

People with LD experience high rates of co-occurring mental illness, with Mazza *et al*^4^ reporting a pooled meta-analysis prevalence estimate of 33.6% (95% confidence interval [CI] = 25.3 to 43.1) for any psychiatric disorder among adults and adolescents with LD (from constituent eligible studies where LD was determined by case register identification or psychiatric interview). Furthermore, adults with LD are at significantly higher risk of having a wide range of physical health conditions, including epilepsy, constipation, visual impairment, hearing impairment, and asthma.^5^ The *Learning from Lives and Deaths: People with a Learning Disability and Autistic People (LeDeR)* report for 2022 describes a median age of death of 62.9 years for both adult males and adult females with LD in England (compared with 86.1 years for adult females and 82.6 years for adult males without LD in the general population),^6^ with 42% of deaths deemed avoidable.^7^

Despite their high level of clinical need, people with LD and/or autistic people experience significant barriers to accessing primary care, including challenges to effective communication with healthcare professionals, a lack of accessible information, fear and embarrassment, long waiting times, and a lack of knowledge of LD among some healthcare professionals.^8^ Such factors can lead to unmet healthcare needs in this patient group, such as reduced uptake of cervical cancer screening.^9^ Furthermore, some people with LD, particularly those with mild LD, may not be known to clinical services, despite potentially having significant healthcare needs.^10^

Under the UK Equality Act 2010,^11^ public sector organisations have a statutory requirement to make reasonable adjustments to ensure that their services are equally accessible to people with LD. Furthermore, these adjustments should be made proactively, rather than simply reacting under circumstances where patients encounter difficulties. There is additionally a legal accessible information standard, which requires NHS and adult social care organisations to provide information in a form that they can understand.^12^ NHS England, a government organisation who provide leadership in the delivery of publicly funded NHS services across England,^13^ cited LD as a priority in their 2019 *NHS Long Term Plan*,^14^ with goals to tackle causes of morbidity and preventable death, and improve understanding of the needs of people with LD across the NHS.

**Table:** How this fits in

Adults with a diagnosis of learning disability are at increased risk for a range of mental and physical health conditions, with a significantly reduced life expectancy compared with the general population. To the authors’ knowledge, this is the largest study of long-term conditions and primary care experiences among adults who report having learning disability. They report heightened rates for a wide range of chronic health conditions and are more likely to express a preference for a particular GP, while being less likely to report confidence and trust in their healthcare professional, or their needs being met.

NHS England commissions the national General Practice Patient Survey (GPPS) to provide evidence to support healthcare improvement for process measures of care quality. It is an annual cross-sectional postal survey of adults registered with a GP practice in England. The questionnaire asked of survey participants covers a wide range of demographic, social, and health-related factors.^15^

Understanding the needs of the population reporting LD, rather than solely relying on those with a clinical diagnosis, is necessary for identifying how current models of health care do and do not meet their needs. In this analysis of the 2022 GPPS the authors aimed to examine the characteristics and primary care experiences of adults reporting LD, compared with those who do not, in England.

## Method

### GPPS 2022 sampling

The 2022 GPPS was conducted by Ipsos MORI, with the sample drawn from patients registered with GP practices in England between 10 January and 11 April 2022.^16^ The sampling frame comprised those aged ≥16 years (hereafter referred to as ‘adults’) with a valid NHS number and continuously registered with an NHS GP practice in England for ≥6 months, with samples from each practice stratified by age, gender, and postcode.^16^ Over 2.47 million individuals were selected at random and had questionnaires mailed to their registered home address; 719 137 questionnaires were completed, representing a 29.1% response rate.^15^^,^^16^ No data were collected on whether the intended addressee or a proxy (such as a carer) completed or supported the sampled patient to complete the questionnaire. Weights were developed by the survey contractor adjusting for sampling design and known patterns of non-response so that the achieved sample was representative of the GP-registered population.^15^^,^^16^ Methodological detail for GPPS 2022, including the questionnaire, is available.^16^ Individual-level GPPS data were provided via a data-sharing agreement with NHS England.

### LD identification

An item on whether the survey responder has LD was first introduced in the 2016 GPPS, as a stand-alone question.^17^ For the 2022 GPPS,^15^ the question asked: ‘Which, if any, of the following long-term conditions do you have?’ with ‘a learning disability’ as one of the response options, a similar approach to that used to identify people with LD in Scotland’s census.^18^ Consequently, participating adults were required to self-report whether or not they think, or know, or believe, or have been told they have a LD. Some responders with a learning difficulty (referred to as a developmental learning disorder in ICD-11),^19^ defined as having *‘a reduced intellectual ability for a specific form of learning’*,^20^ such as seen with dyslexia and dyspraxia, rather than LD, may have erroneously checked this item. There was no objective independent test within the survey to determine where this had occurred, and this issue is discussed further in the section on study limitations in the discussion. A binary coded variable was derived identifying those reporting LD and those who did not. Participants who did not provide a valid response were excluded from all analyses. Written descriptions of the groups reporting LD and not reporting LD are summarised in Box 1.

**Box 1.** Summary of adults reporting LD and not reporting LD^a^

**Adults reporting LD**	**Adults not reporting LD**
Adults with a clinical diagnosis of LD who identify as having LD Adults without a clinical diagnosis of LD who identify as having LD and who would meet diagnostic criteria for LD if subjected to clinical assessment Adults without a clinical diagnosis of LD who identify as having LD and who would not meet diagnostic criteria for LD if subjected to clinical assessment	Adults with a clinical diagnosis of LD who do not identify as having LD Adults without a clinical diagnosis of LD who do not identify as having LD and who would not meet diagnostic criteria for LD if subjected to clinical assessment Adults without a clinical diagnosis of LD who do not identify as having LD and who would meet diagnostic criteria for LD if subjected to clinical assessment

a *Summary developed by the authors of this article. LD = learning disability.*

### Demographic and health survey items

Self-completed social and demographic items covered age, ethnicity, gender, transgender history, sexual identity, religion, caring responsibilities, and smoking status. Socioeconomic items included participant-reported employment status and neighbourhood deprivation based on the Index of Multiple Deprivation (IMD) quintile of the participant’s home address were included in the survey data.

Self-reported chronic health conditions were captured using a multiple-choice question and included: dementia; arthritis/musculoskeletal problems; autism; visual impairment; breathing condition; cancer (last 5 years); hearing impairment; diabetes; heart condition; high blood pressure; kidney or liver disease; mental health condition; neurological condition; stroke (affecting your day-to-day life); and another long-term condition or disability. A subsequent question (within the same survey) on ‘long COVID’ (described as experiencing symptoms >12 weeks after first having COVID-19) was also included in the current analyses.

### Patient experience survey items

The survey also included questions on participants’ self-reported experiences of primary care services, using Likert-scale response options. Items covered five broad domains:

- overall experience;
- before trying to make an appointment;
- access;
- continuity; and
- communication.

Responses were categorised as positive or negative, producing a binary classification in line with the GPPS National Report.^15^ Question wording and categorisation of responses are outlined in Supplementary Table S1.

### Missing data

Participants with missing data for long-term conditions were excluded from the analyses. Models adjusted for age, gender, ethnicity, and deprivation, for which missingness varied between 0.1% and 2.0%; thus, complete case analysis was performed.

### Statistical analysis

All results report unweighted sample counts alongside weighted proportions with 95% CIs. Participant characteristics are described for those reporting LD and those who did not. Weighted percentage point differences are additionally reported, to enable comparisons between adults reporting LD from those who did not.

To examine differences in chronic health conditions between those reporting LD and those who did not, logistic regression models adjusting for age, ethnicity, gender, and area-level deprivation (IMD quintile) were fitted to return adjusted odds ratios (aOR) with 95% CIs and *P*-values. Differences in the occurrence of long-term conditions between the two groups by age were investigated through the incorporation of an interaction effect between age and LD status. The marginal probability of each long-term condition by LD status and age was calculated and are presented graphically.

A similar approach was taken to compare the primary care experiences of adults reporting LD with those who did not. GP practice cluster information was not available; however, robust standard errors were used to allow for some heteroscedasticity (heterogeneity of variance) in patient experiences across GP practices.

### Sensitivity analysis

A sensitivity analysis was performed, which excluded patients who reported having dementia or autism from the analysis of experiences of primary care, as these conditions were the long-term conditions found to have the highest adjusted odds ratios for adults reporting LD compared with those who did not (Table 1). These conditions were only excluded for the sensitivity analyses, but not for other analyses reported in this article.

**Table 1.** Prevalence of long-term health conditions, by whether participant reports LD

**Long-term health condition**	**Total responders (*N* = 623 157)**			**LD (yes) (*N* = 6711)**			**LD (no) (*N* = 616 446)**			**Logistic regression^a^**		
	***n***	**Weighted** %**^b^**	**95% CI**	***n***	**Weighted** %**^b^**	**95% CI**	***n***	**Weighted** %**^b^**	**95% CI**	**aOR**	**95% CI**	***P*-value**
**Alzheimer’s disease or other cause of dementia**	5248	0.6	0.6 to 0.6	217	1.9	1.5 to 2.2	5031	0.6	0.5 to 0.6	9.33	(7.48 to 11.64	<0.001
**Arthritis or ongoing problem with back or joints**	151 982	17.5	17.4 to 17.6	1719	17.1	16.0 to 18.4	150 263	17.5	17.4 to 17.6	2.46	2.23 to 2.72	<0.001
**Autism**	4481	1.4	1.3 to 1.5	1287	25.3	23.7 to 27.0	3194	1.0	0.9 to 1.0	18.44	16.48 to 20.63	<0.001
**Blindness or partial sight**	10 784	1.4	1.3 to 1.4	489	6.0	5.3 to 6.9	10 295	1.3	1.2 to 1.3	8.90	7.66 to 10.36	<0.001
**Breathing condition, such as asthma or COPD**	79 399	11.3	11.2 to 11.4	1414	17.7	16.5 to 19.0	77 985	11.1	11.0 to 11.3	2.05	1.87 to 2.24	<0.001
**Cancer diagnosis or treatment in the last 5 years**	29 032	3.2	3.1 to 3.2	252	2.2	1.8 to 2.6	28 780	3.2	3.1 to 3.2	1.66	1.38 to 2.00	<0.001
**Deafness or hearing loss**	52 501	5.9	5.8 to 5.9	913	10.0	9.0 to 11.2	51 588	5.8	5.7 to 5.9	4.87	4.26 to 5.57	<0.001
**Diabetes**	66 069	7.8	7.7 to 7.8	978	8.7	8.0 to 9.5	65 091	7.7	7.7 to 7.8	2.28	2.06 to 2.54	<0.001
**Heart condition, such as angina or atrial fibrillation**	50 449	5.6	5.5 to 5.6	563	5.8	5.1 to 6.7	49 886	5.6	5.5 to 5.6	2.65	2.24 to 3.13	<0.001
**High blood pressure**	143 318	15.9	15.8 to 16.0	1253	10.8	10.0 to 11.7	142 065	16.0	15.9 to 16.1	1.73	1.56 to 1.93	<0.001
**Kidney or liver disease**	16 317	2.0	2.0 to 2.1	373	3.7	3.3 to 4.3	15 944	2.0	2.0 to 2.0	3.10	2.67 to 3.60	<0.001
**Mental health condition**	60 899	12.3	12.2 to 12.4	2763	41.0	39.3 to 42.7	58 136	11.8	11.7 to 11.9	3.88	3.59 to 4.20	<0.001
**Neurological condition, such as epilepsy**	12 963	2.1	2.0 to 2.2	811	11.4	10.4 to 12.4	12 152	1.9	1.9 to 2.0	6.89	6.18 to 7.67	<0.001
**Stroke which affects your day-to-day life**	7163	0.8	0.8 to 0.9	231	2.2	1.8 to 2.7	6932	0.8	0.8 to 0.8	5.83	4.76 to 7.14	<0.001
**Another long-term condition or disability**	91 286	13.8	13.7 to 14.0	1949	26.3	24.8 to 27.8	89 337	13.6	13.5 to 13.7	2.67	2.46 to 2.89	<0.001
**Long COVID**	24 751	4.8	4.7 to 4.8	371	6.1	5.3 to 7.1	24 380	4.7	4.7 to 4.8	1.19	1.01 to 1.40	0.033

a *Adjusted for age, gender, deprivation, and ethnicity.*

b *Weighted percentages are calculated using survey design and non-response weights by age, gender, geographic location, and GP practice. aOR = adjusted odds ratio. CI = confidence interval. COPD = chronic obstructive pulmonary disease. LD = learning disability.*

In a second sensitivity analysis, the analysis of experiences of primary care were run using different comparator groups, first comparing with those reporting no long-term health conditions and second comparing with those reporting ≥1 other long-term health condition.

## Results

### Frequency of reported LD

A total of 6711 of the 623 157 participants included in the analyses self-reported LD, yielding a weighted proportion estimate of 1.8% (95% CI = 1.7 to 1.9) of the sample. The 70 900 (9.9% of a total of 719 137 participant responses) participants with missing data for long-term conditions were excluded from these analyses, as well as a further 25 080 (3.5% of a total of 719 137 participant responses) participants with missing age, gender, ethnicity, or deprivation information.

### Demographic characteristics

Supplementary Table S2 summarises the demographic characteristics of all survey responders, as well as stratified by self-reported LD status. Adults reporting LD were more likely to describe their gender as male or non-binary, and to describe themselves as gay or lesbian, bisexual, or other. Those reporting LD were also younger (with higher proportions in the 16–24-year and 25–34-year age groups), less likely to identify as being of Asian or Asian British ethnicity, and more likely to identify with no religion.

Adults reporting LD were less likely to report having parental responsibility for a child in their household, and more likely to report having no unpaid caring responsibilities for other people. In terms of employment status, they were less likely to report being in full-time work, and more likely to report being in full-time education, unemployed, or permanently sick or disabled. They were also more likely to be living at an address in the most deprived neighbourhoods.

### Long-term conditions

Table 1 shows the reported occurrence of long-term health conditions and long COVID in adults reporting LD compared with those who do not, after adjusting for age, gender, deprivation, and ethnicity. All 16 conditions had significantly higher odds of being reported by adults reporting LD compared with those who did not so describe themselves.

**Figure 1.**
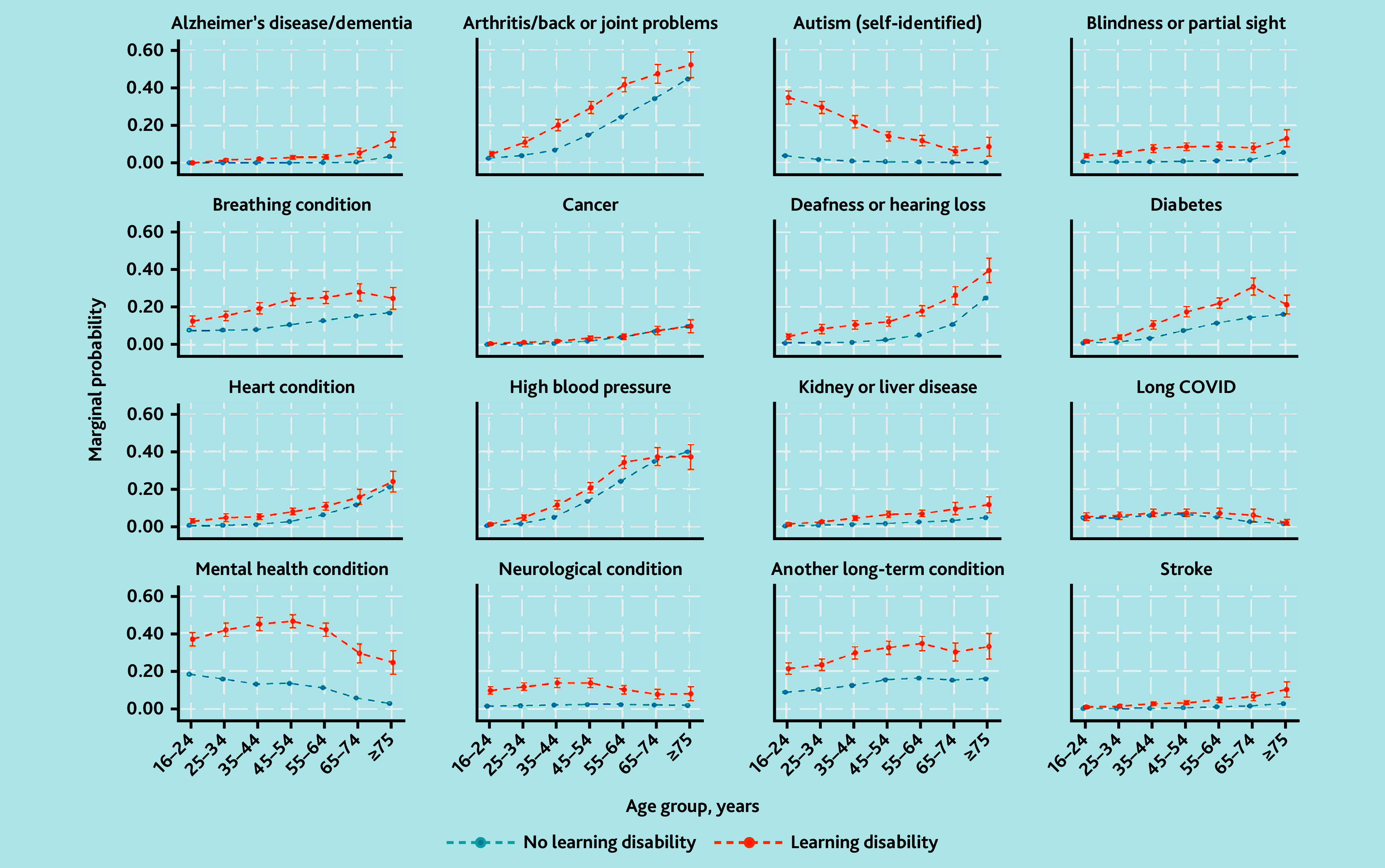
Marginal probability of long-term health condition or disability over age groups, by whether participant reports LD. LD = learning disability.

Figure 1 depicts differences in the marginal probability of long-term conditions between those reporting LD and those who did not by age. The additional likelihood of self-identified autism in adults reporting LD is much greater in younger age groups, and declines with older age groups. Other conditions showed an increased difference in marginal probabilities across middle age groups compared with extreme of age, such as arthritis, breathing conditions, diabetes, and self-reported mental health conditions.

### Experiences of primary care

Table 2 shows the responses to patient experience question items. Although for some question items no significant differences were found, several distinct differences with respect to their experiences of primary care were identified.

**Table 2.** Experience of primary care, by whether participant reports LD

**Experience**	**Total responders (*N* = 623 157)**				**LD (yes) (*N* = 6711)**			**LD (no) (*N* = 616 446)**			**Logistic regression^a^**		

	**Response % ^b^**	***n***	**Weighted % ^c^**	**95% CI**	***n***	**Weighted** %**^c^**	**95% CI**	***n***	**Weighted % ^c^**	**95% CI**	**aOR**	**95% CI**	***P*-value**
**Overall experience**													
Overall positive experience of GP practice	99.4	475 422	72.7	72.5 to 72.9	4769	69.5	67.8 to 71.1	470 653	72.8	72.6 to 72.9	1.07	0.99 to 1.16	0.082
Overall positive experience of making appointment	93.9	355 207	56.4	56.3 to 56.6	3636	55.2	53.4 to 57.1	351 571	56.5	56.3 to 56.7	1.07	1.00 to 1.16	0.067

**Before trying to make an appointment**													
Used an online NHS service	93.1	71 988	16.6	16.5 to 16.8	933	18.1	16.7 to 19.7	71 055	16.6	16.4 to 16.7	0.79	0.72 to 0.88	<0.001
Used a non-NHS online service	93.1	66 050	14.9	14.7 to 15.0	716	13.8	12.6 to 15.1	65 334	14.9	14.7 to 15.0	0.69	0.62 to 0.77	<0.001
Spoke to a pharmacist	93.1	93 091	16.5	16.3 to 16.6	1214	20.0	18.6 to 21.6	91 877	16.4	16.3 to 16.6	1.33	1.20 to 1.46	<0.001
Tried to treat myself	93.1	140 586	26.7	26.6 to 26.9	1402	24.2	22.6 to 25.8	139 184	26.8	26.6 to 27.0	0.79	0.72 to 0.86	<0.001
Called an NHS helpline	93.1	38 711	8.0	7.9 to 8.2	706	11.5	10.4 to 12.8	38 005	8.0	7.9 to 8.1	1.24	1.10 to 1.40	<0.001
Contacted or used another NHS service	93.1	24 744	4.9	4.8 to 5.0	416	7.0	6.2 to 8.0	24 328	4.9	4.8 to 4.9	1.25	1.09 to 1.45	0.002
Asked for advice from friends or family	93.1	96 162	21.4	21.2 to 21.5	1545	29.2	27.5 to 30.9	94 617	21.2	21.1 to 21.4	1.03	0.94 to 1.12	0.534
Tried to get information or advice elsewhere	93.1	50 394	11.1	11.0 to 11.2	568	10.8	9.6 to 12.1	49 826	11.1	11.0 to 11.2	0.76	0.67 to 0.87	<0.001

**Access**													
Easy to use GP practice’s website	54.2	233 960	67.2	67.0 to 67.5	1903	58.1	55.7 to 60.5	232 057	67.4	67.2 to 67.6	0.71	0.65 to 0.79	<0.001
Easy to get through to someone on the phone	95.9	352 018	52.9	52.7 to 53.0	3608	51.8	50.0 to 53.6	348 410	52.9	52.7 to 53.1	1.02	0.95 to 1.10	0.562
Found the receptionists at GP practice helpful	95.8	511 890	82.4	82.2 to 82.5	5218	79.8	78.3 to 81.3	506 672	82.4	82.3 to 82.6	1.10	1.01 to 1.21	0.039
Satisfied with GP appointment times	84.0	314 871	55.3	55.1 to 55.5	3496	57.1	55.2 to 59.0	311 375	55.3	55.1 to 55.5	1.25	1.16 to 1.36	<0.001
Satisfied with appointment offered	83.7	391 447	72.1	71.9 to 72.3	3987	72.8	71.1 to 74.5	387 460	72.1	71.9 to 72.3	1.17	1.07 to 1.28	0.001
In-person appointment at own GP practice^d^	77.4	226 054	46.1	45.9 to 46.3	2229	47.6	45.5 to 49.7	223 825	46.1	45.9 to 46.3	1.04	0.95 to 1.13	0.419

**Continuity**													
Have a preferred GP	93.7	280 260	43.1	42.9 to 43.3	3572	54.2	52.3 to 56.0	276 688	42.9	42.7 to 43.1	2.08	1.93 to 2.25	<0.001
Able to see or speak to preferred GP^e^	38.2	111 203	43.4	43.1 to 43.7	1328	41.7	39.1 to 44.2	109 875	43.5	43.2 to 43.8	0.94	0.85 to 1.05	0.275

**Communication**													
Involved in decisions about care and treatment	83.2	474 164	90.1	90.0 to 90.2	4921	86.9	85.7 to 88.1	469 243	90.2	90.0 to 90.3	0.92	0.83 to 1.03	0.160
Had mental health needs recognised and understood	41.7	215 632	81.0	80.8 to 81.2	3816	79.0	77.3 to 80.6	211 816	81.1	80.8 to 81.3	0.99	0.89 to 1.10	0.859
Confidence and trust in healthcare professional	92.1	541 619	93.3	93.1 to 93.4	5401	90.1	89.0 to 91.2	536 218	93.3	93.2 to 93.4	0.87	0.77 to 0.99	0.030
Needs were met	92.3	534 101	91.1	91.0 to 91.2	5262	87.0	85.7 to 88.1	528 839	91.2	91.1 to 91.3	0.87	0.78 to 0.97	0.015

a *Adjusted for age, gender, deprivation, and ethnicity.*

b *Proportion of participants who provided a response to the survey item.*

c *Weighted percentages are calculated using survey design and non-response weights by age, gender, geographic location, and GP practice.*

d *Base: patient who accepted an appointment the last time they tried to book.*

e *Base: patients with a preferred GP. aOR = adjusted odds ratio. CI = confidence interval. LD = learning disability.*

There were no significant differences between adults reporting LD and those who did not with respect to their overall experience of their GP practice as well as making an appointment.

Prior to making an appointment, adults reporting LD were more likely to report that they had spoken to a pharmacist, called an NHS helpline, and contacted or used another NHS service. However, they were less likely to have used either an NHS or non-NHS online service, tried to treat themselves, or tried to get information or advice elsewhere. No significant difference was identified with respect to asking for advice from friends or family before making an appointment.

For question items on access and continuity, adults reporting LD were more likely to find the receptionists at the GP practice helpful, be satisfied with the GP appointment times, be satisfied with the appointment offered, and have a preferred GP. However, they were less likely to find the GP practice’s website easy to navigate. No significant differences were identified in relation to being offered an in-person appointment at their own GP practice or being able to see or speak to their preferred GP.

For question items on communication, adults reporting LD were less likely to report having confidence and trust in healthcare professionals, and having their needs met. No significant differences were identified with respect to being involved in decisions about care and treatment or having their mental health needs recognised and understood.

After excluding participants reporting autism or Alzheimer’s disease or other cause of dementia within a separate sensitivity analysis, the findings for patient experiences of primary care were unchanged, except a slight reduction in odds of having a preferred GP (aOR 1.90 (1.75 to 2.07) (Supplementary Table S3) compared with aOR 2.08 (1.93 to 2.25) (Table 2). When using different comparator groups, odds of overall positive experiences for adults reporting LD were higher than that for responders with other long-term conditions, whereas measures of communication were worse than that for responders with no long-term conditions (Supplementary Table S4).

## Discussion

### Summary

This study has reported on the characteristics and primary care experiences of adults reporting LD in England. Adults reporting LD are more likely to describe themselves as male, younger, non-religious, have no unpaid caring responsibilities, be in full-time education, unemployed, or permanently sick or disabled, and live in the most deprived neighbourhoods. They also report heightened rates of a wide range of chronic health conditions. With respect to primary care experiences, they are more likely to report difficulties using their GP practice’s website, which provides a barrier to accessing primary care support. They are also more likely to have a preferred GP, although they are less likely to report confidence and trust in their healthcare professional or their needs being met.

### Strengths and limitations

This study benefits from a large sample size of 6711 adults reporting LD, and a comparator group of 616 446 adults completing the same survey questionnaire. The stratified probability approach helps additionally ensure that the study population is representative of adults registered with GP practices across England.

The survey requirement of adults reporting LD themselves is not only a strength but also a limitation because the authors of the current study have no objective test of participants to formally confirm such a diagnosis. Some may have a learning difficulty (referring to a specific domain of reduced intellectual functioning, for example, dyslexia or dyspraxia)^20^ rather than LD, which are terms that are sometimes confused with one another.^21^ However, data obtained on people with LD through such an approach have previously been reported in the research literature,^22^ and reporting of such findings serves to give this marginalised patient group a voice. Furthermore, a formal diagnosis of LD is not a requirement to satisfy the definition of disability under the terms of the UK Equality Act 2010 (although medical evidence of the impact of the person’s impairment is required).^23^

Furthermore, some adults with LD might have required support from a carer (or ‘proxy’) to complete the survey, which could have influenced replies. Although many adults with mild LD (who represent the majority of people with LD)^24^ could complete the survey with carer support, those with moderate-to-profound LD would generally be unlikely to be able to provide meaningful answers to many of the survey question items, even with carer support. This may have led to the LD survey responder population being skewed towards those with milder LD. Another possibility is that, for some adults with moderate-to-profound LD, their surveys may have been completed by their caregiver, and not be necessarily entirely representative of the patients’ own experiences, although carers can express their own views about how they feel the person with LD they care for felt, based on their knowledge of them. This is preferable to the exclusion of such carer responses, which would risk only receiving survey responses relating to adults with milder forms of LD, compromising the generalisability of the data.

The accessibility of surveys for people with LD can be enhanced through multiple approaches, including involving them in question development, pilot testing of questions with a group of people with LD, and adding visual cues to the survey to provide added context.^25^ Furthermore, the list of long-term conditions that were enquired about on the survey were limited, with certain conditions particularly relevant to people with LD, such as attention-deficit hyperactivity disorder and epilepsy, not being included. Additionally, the 2022 GPPS overall had a response rate of 29.1%,^15^ and the characteristics of responders may not be representative of the pool of patients from which they were sampled, although the weighting helps partially address this issue.

### Comparison with existing literature

An analysis of data from Scotland’s census, similarly based on participants’ self-/proxy reporting of LD, previously reported increased representation of people with LD among male and younger age groups, consistent with the findings reported here, although data on other demographic characteristics, such as ethnicity, religion, and sexuality, were not reported.^22^ The elevated rates of chronic health conditions among adults reporting LD are consistent with previous findings, including related to mental illness,^22^^,^^26^ autism,^27^ and physical health conditions.^22^^,^^28^

Previous qualitative research on improving primary care access for people with LD has described primary care interfaces as being *‘misaligned with the needs of people with learning disabilities’*, with a GP commenting on a lack of accessible information online for this patient group.^29^ Development of such accessible information may be most helpful when it is tailored to their individual patient’s needs, rather than developed with all people with LD in mind.^30^ Additionally, such information also needs to be available for people with LD for whom English is not their first language. Furthermore, a literature review^31^ reported that the health information needs of people with LD are being inadequately met. These findings are in line with the current analysis, where adults reporting LD were less likely to report their needs being met and finding the GP practice’s website easy to use. Relatedly, people with LD are less likely to have essential digital skills,^32^^,^^33^ as well as being less likely to use the internet, or own a computer or smartphone.^34^ Thus, there is an increased risk of people with LD having difficulties accessing primary care, particularly for practices where the internet represents the conventional means of booking an appointment.

Additionally, although the GPPS has included a question item relating to having LD for several years, to the best of the authors’ knowledge this is the first journal article reporting national survey data on the primary care experiences of adults reporting LD.

### Implications for research and practice

The survey findings about actions taken by adults reporting LD before making an appointment can inform targeted public health interventions for this group. For example, pharmacists may represent an invaluable point of intervention for adults with LD given their increased tendency to speak to them before making an appointment. There is a need to improve the accessibility of practice websites for adults with LD, perhaps through patient and public involvement in website development and accessibility evaluations. More granular data is required to better understand why adults reporting LD are less likely to have confidence and trust in their healthcare professional and feel that their needs were met. Such approaches could include a more targeted survey specifically for adults reporting LD, as well as semi-structured interviews and qualitative research, with additional carer involvement.

The findings also have implications for primary care practice in England, in relation to the annual LD health checks. This is a government-incentivised scheme, whereby NHS GPs are paid to conduct assessments of people with LD aged ≥14 years, with a view to *‘identifying previously unrecognised health needs, including those associated with life-threatening illnesses’*.^35^ The finding that people with reported LD are more likely to have a preferred GP would support an approach where patients are given flexibility to choose who conducts their annual health check wherever possible. Continuity of care with a preferred GP may lead to greater uptake and engagement in future health checks. Furthermore, public health promotion of annual health checks, both at a local GP practice level, as well as regional and national levels, could be informed by the demographic characteristics of people with LD, such as targeting deprived neighbourhoods, where this patient group are more likely to reside. Additionally, based on the findings of people with LD being more likely to report their needs not being met, the annual health check could itself represent an ideal opportunity to also ensure that areas of priority importance for the patients themselves are identified and addressed wherever possible. However, to provide such adjustments on a national scale, GPs need to be well supported, including having longer consultation times where required,^36^ to provide such a service.

For future versions of the GPPS, an easy-read version of the survey could be considered, with simplified terminology used in the survey questions, and pictures accompanying the questions. However, such an approach may compromise the ability to compare the findings for adults with and without LD (as the question wording would differ) and make year-on-year trend comparisons. Furthermore, there are mixed findings with respect to the accessibility value of adding accompanying illustrations to questions.^37^ It would, however, be helpful to collect further information on LD, including whether the responder has a clinical diagnosis, their severity of LD, and whether their carer assisted them in completing the survey. The person completing the questionnaire should also be asked if they are the person to whom it was addressed, and, if not, their relationship with that person (for example, paid carer or family member).

## Data

Data are not publicly available, and may be obtained through completing a data sharing agreement through liaison with the General Practice Patient Survey team.
